# Extended spectrum beta lactamase producing organisms causing urinary tract infections in Sri Lanka and their antibiotic susceptibility pattern –A hospital based cross sectional study

**DOI:** 10.1186/s12879-017-2250-y

**Published:** 2017-02-10

**Authors:** M. M. P. S. C. Fernando, W. A. N. V. Luke, J. K. N. D. Miththinda, R. D. S. S. Wickramasinghe, B. S. Sebastiampillai, M. P. M. L. Gunathilake, F. H. D. S. Silva, R. Premaratna

**Affiliations:** 1 Professorial Medical Unit, North Colombo Teaching Hospital, Ragama, Sri Lanka; 2 Department of Clinical Pharmacology, Faculty of Medicine, University of Kelaniya, Kelaniya, Sri Lanka; 3 Department of Medicine, Faculty of Medicine, University of Kelaniya, Kelaniya, Sri Lanka

**Keywords:** ESBL producers, Urinary tract infections, Carbapenem resistance

## Abstract

**Background:**

Extended Spectrum Beta- Lactamase producing organisms causing urinary tract infections (ESBL-UTI) are increasing in incidence and pose a major burden to health care. While ESBL producing Klebsiella species seem to account for most nosocomial outbreaks, ESBL-producing *E. coli* have been isolated from both hospitalized and non-hospitalized patients. Although 95-100% ESBL organisms are still considered sensitive to meropenem, rapid emergence of carbapenem resistance has been documented in many countries. The objective of this study was to evaluate urinary tract infections caused by ESBL producers and the antibiotic susceptibility patterns in Sri Lanka.

**Methods:**

Patients with confirmed ESBL-UTI admitted to Professorial Medical Unit, Colombo North Teaching Hospital from January – June 2015 were recruited to the study. Their urine culture and antibiotic susceptibility reports were evaluated after obtaining informed written consent.

**Results:**

Of 61 culture positive ESBL-UTIs, *E. coli* caused 53 (86.8%), followed by Klebsiella in 8 (13.1%).30 (49.1%) had a history of hospitalization within the past three months and included 6/8(75%) of K*lebsiella* UTI and 24/53(45.2%) of *E.coli* UTI. Antibiotic susceptibility of ESBL organisms were; Meropenem 58 (95%), Imipenem 45 (73.7%), Amikacin 37 (60.6%) and Nitrofurantoin 28(45.9%). In 3(4.9%), *E.coli* were resistant to Meropenem. These three patients had received multiple antibiotics including meropenem in the recent past for recurrent UTI.

**Conclusions:**

We observed a higher percentage of *E. coli* over Klebsiella as ESBL producing organisms suggesting most ESBL-UTIs to be community acquired, Carbapenems seem to remain as the first line therapy for majority of ESBL-UTIs in the local setting. However 4.9% prevalence of meropenem resistance is alarming compared to other countries.

Although prior antibiotic utilization and hospitalization may contribute to emergence of ESBL producing Klebsiella and *E.coli* in Sri Lanka, high prevalence of community acquired ESBL-*E. coli* needs further investigations to identify potential causes . Being a third world country with a free health care system, observed alarming rate of carbapenem resistance is likely to add a significant burden to health budget. We feel that treatment of infections in general needs a careful approach adhering to recommended antibiotic guidelines in order to prevent emergence of multi drug resistant organisms.

## Background

Infections caused by extended spectrum beta-lactamase (ESBL)-producing organisms are rising in epidemic proportions and poses a threat and a challenge to clinical practice around the World [1]. Although the exact global prevalence of ESBL producing organisms is not known, certain studies in the Indian subcontinent have found nearly 50% prevalence [2, 3].

ESBLs are a group of plasmid-mediated, diverse, complex and rapidly evolving enzymes which are capable of hydrolyzing penicillins, broad-spectrum cephalosporins and monobactams [1]. While ESBLs are generally derived from TEM and SHV-type enzymes, CTX –M type enzyme isolated from ESBL producers had been recognized as an important subtype leading to multi drug resistance [4]. ESBLs are commonly produced by *E. coli* and Klebsiella species [1]. The plasmids bearing genes-encoding ESBLs also frequently carry genes that encode resistance to other antimicrobial agents, such as aminoglycosides and quinolones [5]. Therefore, the selection of antibacterials against ESBL organisms in clinical practice is often complicated.

Infections caused by ESBL producing organisms range from uncomplicated urinary tract infections (UTIs) to life-threatening sepsis. Fluoroquinolones may be used for the treatment of uncomplicated UTIs when found susceptible, but emerging resistance has limited their role in todays’ clinical practice [5]. Therefore, carbapenems are regarded as the drugs of choice in the treatment of severe infections caused by ESBL-producing organisms [5]. However carbapenem resistance has also been increasingly reported in many countries recently [5]. Therefore, antibiotic therapy of infections caused by ESBL producers including that of UTIs is challenging. The options of antibiotics are very limited, and require long term treatment with novel and costly antibiotics such as Fosfomycin and Colistin. Risk factors for urinary tract infections caused by ESBL producers include recent hospitalizations, recent antibiotic treatment, age over 60 years, diabetes, male gender, recent *Klebsiella pneumoniae* infection, previous use of second or third-generation cephalosporins, quinolones, and penicillins [6].

In Sri Lanka the ESBL producing organisms and their antibiotic susceptibility patterns have not been extensively studied. We conducted a hospital based study in order to identify the ESBL producing organisms and their antibiotic susceptibility patterns using patients diagnosed with ESBL-UTI.

## Methods

**Fig. 1:**
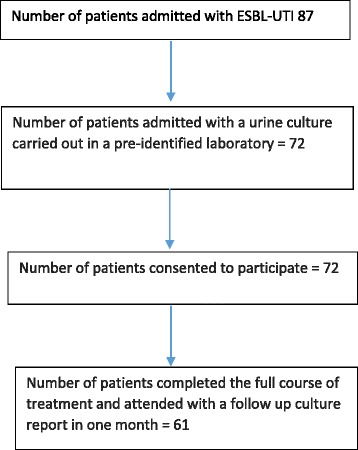
Flow chart on recruitment of study participants

A descriptive cross-sectional study was conducted over a period of six months among adult patients admitted to the Professorial Medical Unit of the Colombo North Teaching Hospital, Ragama, Sri Lanka. Consecutive adult patients who had a culture positive urinary tract infections caused by ESBL producers admitted during the study period fulfilled criteria for selection to the study. (Figure 1: Flow chart on recruitment of study participants) Of them urine cultures that had been performed in five selected private hospital laboratories that maintain quality control and function under the supervision of consultant microbiologists were selected. Patients who consented to provide demographic, clinical and laboratory data were enrolled for the study. In these selected laboratories, ESBL organisms were detected using CLSI or Stokes Disc Diffusion Techniques and carbapenem resistance among ESBL organisms were reported based on the diameter of the zone of inhibition. None of the laboratories routinely performed the modified Hodge test for this purpose. Severity assessment of patients were carried out using clinical parameters (fever >100^0^ F, presence of chills, rigors, renal angle tenderness, reduced urine output) and laboratory parameters (white cell count > 11,000/mm^3^ with neutrophil predominance, high CRP, high serum creatinine and ultrasound evidence of acute parenchymal renal disease or pyelonephritis). Blood cultures were obtained from all severely ill patients based on management protocol for severe pyelonephritis or septicemia. Selections of antibiotics for the treatment of these patients were based on the antibiotic susceptibility patterns documented in the urine culture reports. Ethical clearance was obtained from the Ethics Review Committee, Faculty of Medicine, University of Kelaniya. Informed written consent was obtained from the patients prior to recruitment. The demographic and relevant clinical data was collected and recorded in a pre-tested interviewer administered questionnaire. Information related to past medical history, use of antibiotics and hospitalizations were collected from patients’ follow up medical records. Relevant investigation results and data on treatment during current admission were obtained from ward bed head tickets (in ward patient management records). Data analysis was done using the SPSS software package (IBM Corporation, NY).

## Results

Total of 61 patients consented for the study. Of the 61, 30 (49.1%) were male patients and the mean (SD) age was 64.1(12.6) years. *E.coli* accounted for 53(86.8%) of ESBL UTI’s and Klebsiella was found in the rest 8 (13.1%). (Table 1) Thirty nine (63.9%) had received antibiotics in the past three months (11 were on prophylactic antibiotics);23(37.7%) penicillins (Amoxycillin and co-amoxyclav), 18 (29.5%) 3rd generation cephalosporins, 17 (27.8%) 2nd generation cephalosporins and 18 (29.5%) fluoroquinolones (Ciprofloxacin, Norfloxacin and levofloxacin). They included all 8 (100%) patients who had Klebsiella UTI and 31/53 (58.4%) who had UTI caused by *E. coli.* Of 61 patients, 30 (49.1%) had a history of hospitalization within the past three months and included 6/8(75%) of Klebsiella UTI and 24/53 (45.2%) of *E.coli* UTI (Table 2). Of the study population, 28 patients had blood cultures performed and of which 8 were bacteremic and had the same organism found in urine grown in the blood cultures.

**Table 1:** Characteristics of the study population

Gender	Male 30 (49.1%)
	Female 31 (50,8%)
Isolated organism	*E coli* 53 (86.8%)
	Klebsiella 8 (13.1%)
Co-morbidities	
Diabetes	54 (88.5%)
Hypertension	38 (62.2%)
Bronchial Asthma	8 (13.1%)
Chronic Liver Disease	10 (16.3%)
Renal Stones	3 (4.9%)
USS findings	
Acute pyelonephritis	21 (34.4%)
Chronic Kidney Disease	19 (31.1%)
Hydronephrosis	4 (6.5%)
Hydroureter	4 (6.5%)
Prostatomegaly	4 (6.5%)

**Table 2:** Associations with ESBL UITs

Associated factors	Yes (%)	No (%)
On Prophylactic Antibiotics	11 (18.0%)	50 (81.9%)
Hospitalization in last 3 months	30 (49.1%)	31 (50.8%)
Antibiotic treatment in last 3 months	39 (63.9%)	22 (36.0%)
Previous use of penicillin (Amoxycillin/co-amoxyclav)	23 (37.7%)	38 (62.2%)
Previous use of fluoroquinolones (Ciprofloxacin/Levofloxacin)	18 (29.5%)	43 (70.4%)
Previous use of 2G cephalosporins	17 (27.8%)	44 (72.1%)
Previous use of 3G cephalosporins	18 (29.5%)	43 (70.4%)

With regards to antibiotic susceptibility (Table 3), majority of ESBL organisms were susceptible to carbapenems; 58(95%) to Meropenem and 45(73.7%) to Imipenem, and 37(60.6%) were sensitive to Amikacin and 28(45.9%) to Nitrofurantoin. Therefore, resistance to Meropenem was found in 3 (4.9%) and to Imipenem in 16(26.2%). The three patients who had meropenem resistance had previously received multiple antibiotics including carbapenems for recurrent urinary tract infections and all three of them had diabetes. Two of these isolates were susceptible to amikacin and the other showed susceptibility to Piparacillin-tazobactum. All these patients were treated with respective antibiotics until they had a normal CRP level and required treatment for at least 10-14 days. All study participants had a repeat urine culture one month after the completion of treatment and remained negative for any growth.

**Table 3:** Antibiotic sensitivity pattern

Antibiotic	Yes	No
Meropenem	58 (96.1%)	3 (4.9%)
Imipenum	45 (73.7%)	16 (26.2%)
Nitrofurantoin	28 (45.9%)	33 (54%)
Amikacin	37 (60.6%)	24 (39.3%)
Gentamycin	30 (49.1%)	31 (50.8%)
Ceftrioxone	0	61 (100%)
Ceftazidime	0	61 (100%)
Ciprofloxacin	6 (9.8%)	55 (90.1%)
Piparacillin-tazobactum	12 (19.6%)	49 (80.3%)

## Discussion

In this preliminary hospital based study, our objective was to identify the organisms causing ESBL-UTIs and their antibiotic susceptibility pattern. Therefore our study does not address issues such as incidence and prevalence of ESBL-UTI in the country. All of these patients had a special reference for admission to the unit, mainly based on non-availability or non- affordability of expensive antibiotics or were considered too complicated for the management in peripheral hospitals.

In this study, majority (86.2%) of the ESBL producing isolates were *E.coli* and only 13.8% were Klebsiella. Similar pattern was found in South India in 2010 and a reversed pattern was found in North India in 2013 [3, 7]. Although we observed a higher percentage of *E. coli* over Klebsiella as ESBL producing organisms, it is difficult to conclude that most ESBLs in Sri Lanka are due to *E. coli*. This is because this study has a patient selection bias. Furthermore, ESBL-producing Klebsiella species are considered responsible for most nosocomial outbreaks and they are usually clonal and the strains are known to spread mainly through cross-transmission [2]. There is no evidence that hospital-acquired ESBL-producing *Klebsiellae* are decreasing in importance. Data from the Centers for Disease Control and Prevention show 47% increase of *Klebsiella pneumoniae* isolates from United States intensive care units in 2003 compared with the preceding 5 years [8]. On the other hand, an increase in the number of ESBL-producing *E. coli* is being described in several parts of the world [9–12]. In contrast to ESBL Klebsiella infections, many of the ESBL-producing *E. coli* have been isolated from non-hospitalized patients [9–12], and were less frequently clonally related and found to produce CTX-M enzymes [9–12]. These chromosomally encoded enzymes were found in some environmental bacteria, such as *Kluyvera* species [13], that colonize in farm animals [14, 15], and subsequently in a significant proportion of people in the community [16, 17]. In a study carried out in Netherlands ESBL producing *E. coli* were found in meat and poultry and were similar to strains isolated in rectal swabs and blood cultures of patients with ESBL sepsis, suggesting transmission of ESBL through the food chain [18]. Furthermore, some patients with infections caused by ESBL-producing Enterobacteriaceae did not have any previous significant health care contact suggesting they acquired ESBL producing *E coli* in the community. Similarly, in our study, we found 75% patients with Klebsiella ESBL had hospitalization and 100% of them were treated with an antibiotic during past three months compared to only 49% with *E coli* UTI had hospital admissions and 58% received antibiotics. This may suggest that patients with Klebsiella ESBL would have been nosocomial in origin and majority of *E. coli* would have been community acquired. However, this speculation needs confirmation by genetic studies.

In this cohort of patients with ESBL UTIs, 39 (63.9%) had received antibiotics during the three months prior to admission and out of the antibiotics received, penicillin group (amoxicillin and co-amoxyclav) was the commonest followed by 2nd generation cephalosporins, 3rd generation cephalosporins and fluoroquinolones (ciprofloxacin, levofloxacin and norfloxacin) similar to that had been documented in other studies [2, 19]. Furthermore, 11(18%) were on prophylactic antibiotics for recurrent urinary tract infections and they developed ESBL-UTI while on antibiotics. Today, use of prophylactic antibiotics in recurrent urinary tract infections is no longer recommended as it enhances emergence of resistant strains [20]. Although whether use of prophylactic antibiotic per se, is a risk factor for emergence of ESBL producers is not clear [21], past use of antibiotics has been previously described in association with emergence of ESBL producers [6, 22]. Furthermore, inappropriate use of antimicrobials has been shown to play a pivotal role in the emergence of multi drug resistant organisms. Selection of resistant forms can occur during or after such antimicrobial treatment. In addition, surface antibacterials that are used for disinfection of many household products may play a role in development of antibacterial resistance [23]. Therefore clinicians should ensure the use of appropriate antibiotics for recommended periods in adequate doses in order to prevent emergence of multidrug resistant organisms such as ESBLs.

Furthermore, hospitals should implicate strategies to minimize the spread of ESBL producing organisms by observing universal precautions and minimizing contact among hospitalized patients [24]. This might reduce the spread of ESBL producing organisms in the community. Siegel *et al* recommended that patients infected with multi drug resistant organisms should have restricted contact with other patients [24]. Therefore, although it can be controversial, early discharge of not so seriously ill patients, such as those with ESBL UTIs, with view to home based treatment with potent and effective antibiotics which can be introduced once a day such as Ertapenem [25] or aminoglycosides such as amikacin may be considered in order to prevent spread of ESBL organisms within institutions. Furthermore, adherence to recommended hand washing techniques or use of hand rubs may help to prevent transmission of these infections from one patient to the other [26].

The carbapenems (imipenem, meropenem, ertapenem, doripenem) are still the first choice of treatment for serious infections with ESBL-producing *E. coli* and *K. pneumoniae*. It has been reported that >98% of the ESBL-producing *E. coli*, *K. pneumonia* and *P. mirabilis* are still susceptible to these drugs [27]. But with the emergence of the carbapenem-resistant Enterobacteriaceae, some older drugs were found effective against ESBL-producing *E. coli* or *K. pneumonia* infections. Fosfomycin was reported of having in vitro activity against the ESBL-producing *E. coli* or *K. pneumoniae*. In Hong Kong, most of the ESBL-producing *E. coli* isolates were reported to be sensitive to fosfomycin [28]. Colistin, Tigecycline, Polymyxins and some aminoglycosides are considered effective in the treatment of carbapenem resistant organisms [27]. The role of aminoglycosides should not be forgotten as some species will be sensitive and respond to aminoglycoside therapy. In a Spanish study published in 2014, 50 cases of Carbapenem resistant Klebsiella infections were treated with aminoglycosides showing a statistically significant reduction in mortality [29]. The role of piperacillin-tazobactam (PTZ) for patients infected with ESBL-producing pathogens remains unclear. Although ESBLs are generally inhibited by tazobactam, many organisms produce multiple ESBLs simultaneously, which may reduce the effectiveness of PTZ [30]. In a study done by Tamma et al in 2015 found PTZ to be inferior to carbapenem therapy for the treatment of ESBL bacteremia and suggested early carbapenem therapy for patients at high risk of invasive ESBL infections [31].

In this study, 95% of ESBL organisms were sensitive to meropenem. However one crucial finding of this preliminary study was the3/61 (4.9%) prevalence of meropenem resistance among ESBL organisms. All these three patients were diabetics and had a history of recurrent urinary tract infections with multiple hospitalizations and received multiple antibiotics including meropenem. Although literature on carbapenem resistant ESBL producers is limited, available regional studies demonstrate substantially lower rates of carbapenem resistance. No carbapenem resistance has been documented in India among 167 patients in 2014 [19] and in Bangladesh among 115 patients in 2008 [32]. However, 0.4% carbapenem resistance has been documented in Pakistan in 2007 [33].

## Conclusions

*E. coli* and Klebsiella were found to be the main ESBL-UTI among the patients referred for further management in the study setting and occurrence of carbapenem resistance was observed within them. Although most ESBL-UTIs had an association with past hospitalization and antibiotic use similar to that is documented in other countries, its significance needs to be confirmed with a proper control group. Occurrence of community acquired ESBL-UTI needs further study to identify the likely reasons and the sources of such infections. Sri Lanka, being a third world country with a free health care system, presence of infections caused by ESBL producers and occurrence of carbapenem resistance among them is likely to add a significant burden to health budget. We feel that treatment of infections in general needs a careful approach and stress upon the value of adherence to recommended antibiotic guidelines in order to prevent emergence of multi drug resistant organisms. Furthermore, antibiotics for meropenem resistant ESBL producers such as Fosfomycin and Colistins should be made available while reserving them only for the treatment of life threatening infections caused by ESBL producers.

## Abbreviations

ESBL: extended spectrum Beta-lactamase
UTI: urinary tract infection
